# Idiopathic Intussusception in Infants and Children: Different Outcomes in Relation to Interventions

**DOI:** 10.7759/cureus.48026

**Published:** 2023-10-31

**Authors:** Mohammad Alnamshan, Dana Almatroudi, Dana ALmutairi, Nouf A Almagushi, Leen Almadhi, Afnan M Alenazi

**Affiliations:** 1 Pediatric Surgery, King Abdulaziz Medical City, Riyadh, SAU; 2 College of Medicine, King Saud Bin Abdulaziz University for Health Sciences, Riyadh, SAU

**Keywords:** pediatric bowel emergency, surgical management of intussusception, pneumatic intussusception reduction, idiopathic intussusception, pediatric intussusception

## Abstract

Background

Intussusception is a pediatric emergency causing bowel obstruction that can progress to gangrene or perforation. Patients usually present with vomiting, abdominal discomfort or pain, or rectal bleeding. Specialized infant and child care is important to detect and manage such cases.

Methodology

This retrospective, cross-sectional study analyzed 45 cases of idiopathic pediatric intussusception presented to and managed by specialized pediatric healthcare services over 12 years. The medical records of children who presented with idiopathic intussusception from January 2010 to December 2022 at King Fahad National Guard Hospital, Riyadh, Kingdom of Saudi Arabia were reviewed. The data obtained included age, sex, clinical presentation, symptom duration, diagnostic investigations, mode of treatment, length of hospital stay, and outcomes.

Results

A total of 45 children were included (25 male, 20 female). The median age was 10 months ranging between five and eight months. The majority presented with abdominal pain or colic (78%), vomiting (76%), and rectal bleeding (47%). The diagnosis was done by an ultrasound preceded by pneumatic enema reduction that was successful in 33 (80%) children. Only four (9%) children underwent surgery as initial management. Ileocolic intussusception (73%) was the most prevalent, followed by colicolic (18%) and ilioiliac (10%). Among the children who underwent surgical reduction, 11 (92%) underwent laparotomy reduction. In total, 11 children underwent surgical reduction as well as an appendectomy, and four children required bowel resection. Only two children developed perforation, and recurrence occurred in two other children. The mean duration of symptoms before presentation was 46.73 hours, and the mean hospital stay was 3.4 days for all cases. Rectal bleeding was a predicting factor for surgical reduction.

Conclusions

Ileocolic was the most common site of intussusception. Abdominal pain, vomiting, and rectal bleeding were frequently seen on presentation. In addition, surgical reduction was associated with rectal bleeding. However, pneumatic reduction was successful in 80% of the cases. Unlike the previous study, this study reports fewer children requiring surgical intervention as well as lower hospital stay duration. Thus, this study emphasizes the importance of specialized pediatric services to enhance outcomes.

## Introduction

Intussusception is a life-threatening condition that causes strangulating bowel obstruction which can progress to gangrene and perforation [1]. The term intussusception refers to the invasion of one intestinal segment into a bowel segment that is next to it [2]. Although most patients present with the classical triad of vomiting, abdominal discomfort, and rectal bleeding, few patients also complain of rectal mass prolapse, sleeping infants, or signs of other underlying disorders [3]. The pediatric age group that is frequently diagnosed with intussusception includes children between the ages of four months and two years, with a peak in incidence between four and nine months of age [4]. The site of intussusception may be cecocolic, colocolic, jejunojejunal, or ileoileal; however, the most prevalent site of intussusception in pediatric patients is ileocolic [5]. Moreover, it can be classified into primary or secondary intussusception [6]. Primary intussusception is idiopathic and accounts for 90% of cases in the pediatric age group, whereas secondary intussusception, which is due to pathological lead points such as the appendix, Meckel’s diverticulum, intestinal polyp, intestinal lymphoma, and solid bowel lesions, is rarely encountered [6-8]. Idiopathic ileocolic intussusception is considered the most frequent type of intussusception and non-operative reduction with pneumatic and/or hydrostatic enemas is the usual treatment [2]. Once intussusception is clinically suspected it must be confirmed by ultrasonography where pathognomonic signs such as the “target sign” and “sleeve sign” on cross and vertical sections, respectively, are seen [4,9,10]. The patient’s condition determines the management and treatment. Initial non-operative reduction is attempted among children who are in good overall health under constant ultrasonographic monitoring [4]. Even though air enema reduction is proven to be superior to water-soluble contrast enema reduction, both are used alternatively as first-line treatment for idiopathic intussusception in infants and children after appropriate stabilization [11-13]. If a radiological contraindication is present, such as severe dehydration and/or septic shock, or in case of failure of non-operative attempts, urgent surgical reduction is the final resort [4].

Although intussusception is an emergency and requires immediate intervention, it is not a common phenomenon with a mean incidence of 74 per 100,000 globally [7]. Diagnosis of intussusception is done radiologically in North America, Asia, Europe, Oceania, Eastern Mediterranean, and Central and South America [7]. In Africa, intussusception is mostly diagnosed by clinical findings or during surgery [7]. Treatment is mostly done through radiological reduction, except in Africa and Central and South America where surgery is the most frequent treatment [7]. Locally, a study conducted in Aseer Central Hospital (ACH) reviewed 34 idiopathic intussusception cases in infants and children less than eight years old between 1993 and 2000 [14]. It was found that 100% of cases presented with vomiting and 91% had bleeding per rectum [14]. Unexpectedly, 76% of ACH patients needed an exploratory laparotomy for manual reduction, with six patients undergoing bowel reduction [14]. In a study conducted at King Fahad National Guard Hospital (KFNGH) by Crankson et al., 33 infants and children who were diagnosed with idiopathic intussusception and managed between 1986 and 2000 were reviewed [5]. In the study, intussusception occurred in 90% of infants less than 12 months old [5]. Most (81%) presented with bleeding per rectum, and only 43% presented with the classical triad (abdominal pain, abdominal mass, and rectal bleeding) [5]. Unlike the ACH study, 56% of KFNGH cases were successfully managed with radiological reduction [5]. However, the current outcomes of idiopathic intussusception in relation to therapeutic interventions in Saudi Arabia are unknown. Therefore, this retrospective study aimed to review the outcomes in relation to interventions of pediatric idiopathic intussusception cases managed in King Abdulaziz Medical City from January 2010 to December 2022 to compare it with the findings of the previous study reported by Crankson et al.

## Materials and methods

Study design

This retrospective study assessed the different outcomes of different interventions performed for idiopathic intussusception at King Fahad Medical City (KFMC) which includes King Abdulaziz Medical City (KAMC) and King Abdullah Specialized Children Hospital (KASCH), Riyadh, Kingdom of Saudi Arabia over a 12-year period from January 2010 through December 2022. Both hospitals provide specialized pediatric healthcare. KAMC had a specialized pediatric department for each specialty (emergency, radiology, anesthesia, and pediatric surgery) until KASCH opened in 2016. This study was conducted at the KASCH Pediatric Surgery Department after obtaining ethical approval (approval number: IRB/0059/23). The study included 45 pediatric patients who were diagnosed and managed for idiopathic intussusception at KFMC. Patients with secondary intussusception or transient intussusception were excluded. After obtaining history and conducting physical examinations, all patients were stabilized and sent for abdominal plain radiographs as initial imaging. The diagnostic imaging was done by ultrasound. All cases were treated by pneumatic reduction if laparotomy had failed or laparoscopic reduction was done.

Data collection

The data were collected using a data collection sheet which was divided into seven categories (i.e., patient information, clinical presentation, site of intussusception, radiograph findings, diagnostic modalities, type and mode of therapeutic intervention, and outcomes of management). Data from before 2016 were collected through the paper medical records and after 2016 were gathered through the medical record system BestCare. The data were reviewed on three separate occasions by the authors to ensure accuracy. Only authors had access to the patient data, and patient personal information was kept confidential.

Data management and analysis

After data collection, each variable was coded into serial numbers using Microsoft Excel (Microsoft Corp., Redmond, WA, USA). SPSS version 26 software (IBM Corp., Armonk, NY, USA) was used for data analysis. Numerical variables such as age and length of stay were reported as medians and ranges. To determine the association between two numerical variables, Student’s t-test was used. Categorical variables such as gender and type of intervention were reported as percentages and frequencies. The chi-square test was used to determine the association between two categorical variables. To confirm the findings of the chi-square test, a logistic regression test was used to measure the correlation between outcomes and different variables. All statistical tests were considered significant at p-values of less than 0.05.

## Results

Of the 45 children, 25 were male and 20 were female. The median age was 10 months ranging between five and eight months. The clinical features included abdominal pain or colic (78%), vomiting (76%), and rectal bleeding (47%) (Table 1). After initial resuscitation, the diagnoses were made by an ultrasound preceded by pneumatic enema reduction which was successful in 33 (80%) children. Only four (9%) children underwent surgery as the initial management. Regarding the site of the intussusception, ileocolic intussusception (73%) was the most prevalent, followed by colicolic (18%) and ilioiliac (10%). Among the children who underwent surgical reduction, 11 (92%) had undergone laparotomy, and only one child underwent a laparoscopic reduction. Overall, 11 children underwent surgical reduction as well as an appendectomy, and four children required bowel resection. Only one child developed a wound infection; however, he was in sepsis before surgery. Two children developed perforation, and recurrence occurred in two other children. The mean duration of symptoms before presentation was 46.73 hours, and the mean of hospital stay was 3.4 days for all cases. Rectal bleeding was found to be a predicting factor for surgical reduction (Table 2).

**Table 1:** Clinical features on presentation.

Features	N (%)
Fever	8 (18)
Rectal bleeding	21 (47)
Vomiting	34 (76)
Abdominal pain/Colic	35 (78)
Abdominal mass	5 (11)
Diarrhea	17 (38)

**Table 2:** Factors associated with surgical reduction.

Variables	Odds ratio	Significance
Gender	4.21	0.1
Age	0.95	0.2
Duration of symptoms (hours)	1.01	0.52
Fever	0.62	0.7
Rectal bleeding	0.12	0.02
Vomiting	0.17	0.16
Abdominal pain	0.66	0.67
Diarrhea	3	0.6

## Discussion

International studies

China

According to a Chinese study, 31 (3.40%) episodes were determined to have spontaneously reduced, and five patients (0.7%) underwent surgery as a result of the failure of air enemas or B-USGHE [1]. Similarly, our study found that after the initial resuscitation, the diagnoses were made by ultrasound, followed by a successful pneumatic enema reduction in 33 (73%) children. Four (9%) children underwent surgery as the initial treatment. Another study conducted at the University College Hospital, Ibadan, Nigeria reported a male-to-female ratio of 1.8:1, with a mean age at presentation of 13.4 months. The majority of patients (36.4%) presented between two and three days following the onset of symptoms, with 14 (25.5%) cases presenting during the first 24 hours. Moreover, the average length of stay in the hospital was 12.1 days (range = 3-60 days) [2]. The median age in our study ranged from five to eight months, with 10 months being the closest range. The mean hospital stay across all cases was 3.4 days, and the average number of hours of symptoms before presentation was 46.73 hours [15].

Iraq

In a study by Khaleel et al. from Iraq, the mean age of presentation was six months, similar to this study in which age mostly ranged between five and eight months. Moreover, the most common sign was abdominal pain 55 (96.5%), as observed in this study as well (78%). Furthermore, ileocolic intussusception was most frequently encountered in both Iraq (44, 77.2%) and in our study (73%). In addition, the mean of hospital stay in this study was 3.4 days for all cases, while in the study by Khaleel et al., it ranged between two and seven days. Cases of wound infection in the study by Khaleel et al. were higher at seven (12.3%) cases [16].

Coimbra, Portugal

This study was conducted in a children’s specialized hospital in Coimbra from June 1977 to May 1990. It included 233 patients, with males accounting for 66% of cases. Similar to our study, the most common site was iliocecocolic intussusception in 87% of children who presented at less than one year of age. Moreover, on presentation, abdominal pain (88%) and vomiting (81%) were the most common features. Overall, 94% of children underwent barium enema for diagnostic and therapeutic purposes. Surprisingly, only 54% underwent a successful non-operative reduction, and 121 patients underwent surgical reduction. These outcomes could be a result of many different factors as this study was conducted on a larger number of cases and was conducted before 1990, especially as intussusception management guidelines have changed since then. However, the results compared to other studies that were conducted at general hospitals in the same time period. This finding supports this paper’s hypothesis that pediatric healthcare services are better provided by specialized children’s health workers [17].

National studies

Riyadh

In a previous Saudi study by Crankson et al., the mean age was 8.4 months ranging from five hours to 36 months; however, in our study, the median age was 10 months mostly ranging between five and eight months. Furthermore, the clinical features included abdominal pain or colic (65%) and vomiting (78%). In contrast, in this study, abdominal pain or colic was seen in 78% and vomiting in 76%. Moreover, in the previous study, the diagnosis was made by a plain abdominal radiograph, followed by a contrast enema reduction, which was successful in only 56% of cases. In our study, diagnoses were made by ultrasound preceded by pneumatic enema reduction which was successful in 73% of cases. In the study by Crankson et al., the most common site of intussusception was in the transverse colon (70%) and the least common site was the ascending colon (3%), in contrast to our study where it was ileocolic (73%) and ilioiliac (10%), respectively. In our study, the mean duration of symptoms before presentation was 46.73 hours and the mean hospital stay was 3.4 days for all cases. In the previous study, the mean duration of symptoms was 33 hours and the mean hospital stay was 2.4 days and 6 days for successful enema reduction and laparotomy cases, respectively [5] (Table 3).

**Table 3:** Comparison between a previous study and our study outcomes.

Findings	Crankson et al. [5] (1984–2000)	Our study (2010–2022)
Demographic data		
Number of participants	33 patients	45 patients
Male gender	21 (64%)	25 (56%)
Age (less than 12 months)	31 (94%)	27 (60%)
Features on presentation		
Abdominal pain	24 (65%)	35 (78%)
Vomiting	39 (78%)	34 (76%)
Rectal bleeding	30 (81%)	21 (47%)
Abdominal mass	23 (62%)	5 (11%)
Diarrhea	13 (35%)	17 (38%)
Healthcare outcomes		
Successful enema reduction	20 (56%)	33 (80%)
Length of stay post-successful pneumatic reduction (mean)	2.4 days	1.8 days

Aseer

A study done in Abha at ACH included 34 children, 21 (61%) of whom were male. Likewise, in this study, 25 (56%) children were male. The mean age was 10.86 months in the ACH study compared to a median age of 10 months in this study. In contrast to our study, Al-Malki et al. reported that the most common presentation was vomiting (100%) exhibited by all patients, followed by rectal bleeding (91%), and abdominal pain (82%). Complementary to our study, the most common site of intussusception was iliocolic with 19 (56%) patients. Regarding the diagnostic modalities, a diagnostic barium enema was used in 28 (82.3%) patients with a success rate of 29%. In comparison, our study used an ultrasound with a higher success rate (73%) [14].

## Conclusions

Intussusception mostly occurred in the ileocolic region. Abdominal pain, vomiting, and rectal bleeding were the most common presentations. Pneumatic enema was successful in 80% of cases. In addition to being a common presentation, rectal bleeding was found to be associated with surgical reduction. Compared to the previous study, although the presentation in both time periods was similar, this study reported fewer children requiring surgical intervention after pneumatic reduction, as well as lower hospital stay duration, highlighting the importance of providing specialized pediatric healthcare services for better results. However, this could be a result of a delay in surgical intervention and repetition of pneumatic reduction when the patient was stable, as was recommended by the previous study. Moreover, this study is limited to KFMC cases only, thus we recommend more studies in different centers with bigger populations.
